# Caudal Regression Syndrome

**DOI:** 10.3390/children7110211

**Published:** 2020-11-04

**Authors:** Ranjit I. Kylat, Mohammad Bader

**Affiliations:** Department of Pediatrics, University of Arizona, College of Medicine, Tucson, AZ 85724, USA; mbader@arizona.edu

**Keywords:** caudal dysgenesis syndrome, caudal regression syndrome, sirenomelia

## Abstract

Caudal Regression Syndrome (CRS) or Caudal dysgenesis syndrome (CDS) is characterized by maldevelopment of the caudal half of the body with variable involvement of the gastrointestinal, genitourinary, skeletal, and nervous systems. CRS affects 1–3 newborn infants per 100,000 live births. The prevalence in infants of diabetic mothers is reported at 1 in 350 live births which includes all the variants. A related condition is sirenomelia sequence or mermaid syndrome or symmelia and is characterized by fusion of the legs and a variable combination of the other abnormalities. The Currarino triad is a related anomaly that includes anorectal atresia, coccygeal and partial sacral agenesis, and a pre-sacral lesion such as anterior meningocele, lipoma or dermoid cyst. A multidisciplinary management approach is needed that includes rehabilitative services, and patients need a staged surgical approach.

## 1. Introduction

Caudal Regression Syndrome (CRS) or Caudal dysgenesis syndrome (CDS) is a rare disorder and characterized by maldevelopment of the caudal half of the body with variable involvement of the gastrointestinal, genitourinary, skeletal and nervous systems [1,2,3,4]. It includes a wide range of congenital anomalies affecting the caudal spine and spinal cord, the hindgut, the urogenital system and the lower limbs, and the spectrum of its severity is variable. CRS is reported to affect 1–3 newborn infants per 100,000 live births among the general population. The prevalence rate is much higher in infants of diabetic mothers. A multidisciplinary management approach is needed, and many patients need a staged surgical approach. Herein, we describe a patient with CRS.

## 2. Case Presentation

This 30-week gestation female infant was born via vaginal delivery to a healthy non-diabetic 18-year-old primigravida female. Prenatal ultrasound revealed relatively hypoplastic lower limbs with bilateral clubfoot and low vertebral and sacral anomalies. There was also polyhydramnios and there was suspicion of a form of CRS based on lower limb and vertebral anomalies. Prenatal laboratory tests, including cell-free fetal DNA, were all normal except for her rubella nonimmune status. The family declined to pursue amniocentesis. There was no exposure to teratogens, illicit drugs or alcohol. There was no significant family history forthcoming and no consanguinity. There were no glycemic abnormalities during pregnancy. Her mother was admitted a week prior to birth for preterm labor and received two doses of betamethasone and appropriate antibiotics.

**Figure 1:**
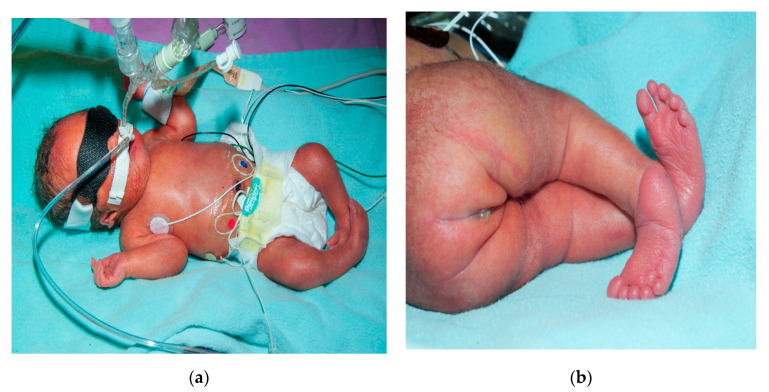
Image depicting club foot, ear, and sacral region anomaly (**a**,**b**).

**Figure 2:**
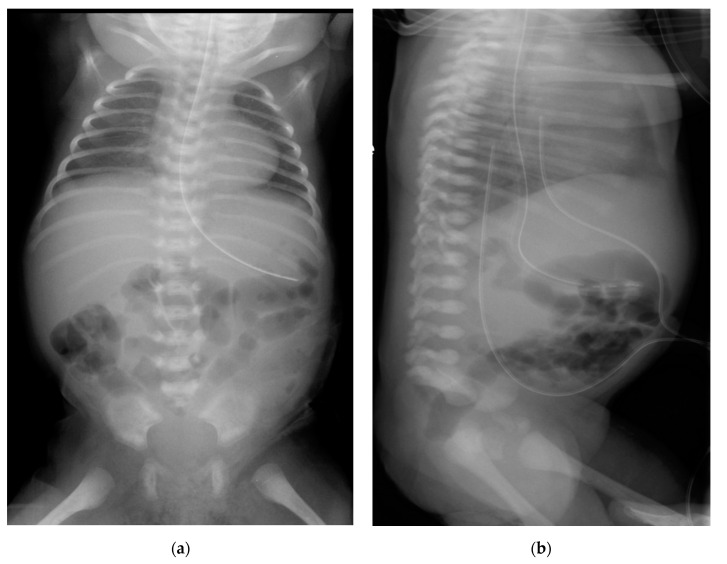
Plain (anteroposterior (**a**) and lateral (**b**)) radiograph of chest and abdomen showing absent sacrum. Both the umbilical arterial and venous catheters on the lateral view were not appropriately positioned but was pulled back to optimal site.

**Figure 3:**
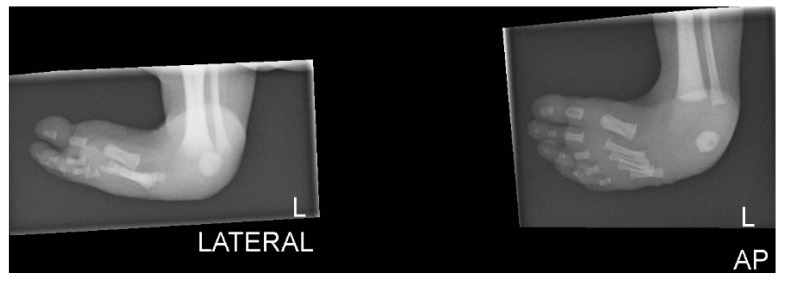
Plain lateral radiograph of foot showing club foot.

**Figure 4:**
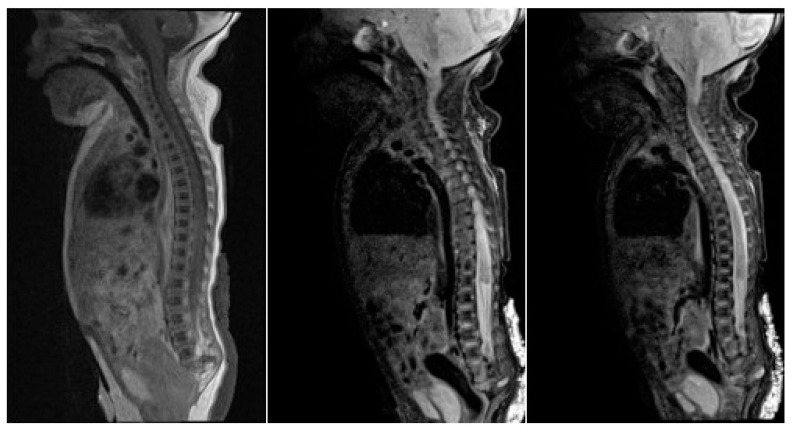
Sagittal MR images show vertebrae down to roughly L5 with no developed sacral elements. The conus medullaris is high at T11, with a truncated, inferior tip.

**Figure 5:**
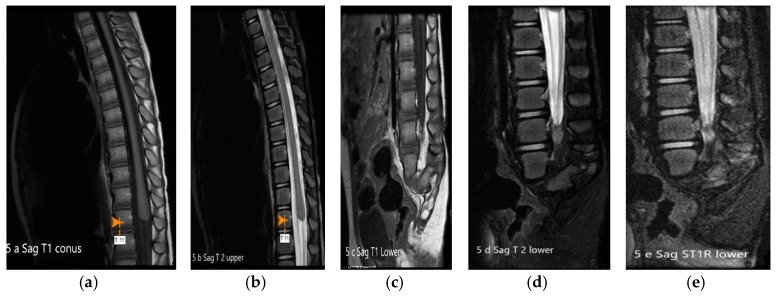
(**a**–**e**): MRI of spine T1, T2, T1 FS and T1 STIR images: The conus medullaris is truncated and nodular at the level of intrapedicular T11 with rudimentary dysgenetic sacrum and sagittal cleft in the median sacrum with rudimentary apophysis. Thickened cauda equine is seen. FS—fat suppression. STIR—short T 1 inversion recovery.

On initial assessment infant was noted to have underdeveloped lower limbs, bilateral club foot, imperforate anus and absent sacrum. (Figure 1, Figure 2 and Figure 3) Other abnormal features included low set ears, depressed nasal bridge and high arched palate. A three-vessel umbilical cord was present. Spinal ultrasound (US) revealed absence of the sacrum with the conus having a blunt end at the tip at the T12-L1 disc space. Renal US showed multicystic dyplastic right kidney, duplicated right and left renal collecting system, and distended right ureter. Head US revealed premature gyral and sulcal pattern that was, however, appropriate for the gestational age. Magnetic resonance imaging (MRI) of the spine at a month of age revealed a rudimentary sacrum, absence of coccyx and blunting of spinal cord at T 11 and features suggesting a type 1 CRS (Figure 4). An echocardiogram revealed small mid-muscular VSD with left to right shunting. A karyotype and chromosomal microarray did not reveal any abnormalities. She underwent a colostomy and mucus fistula creation within 2 days when a rectovaginal fistula was also noticed. Prophylactic antibiotics was initiated due to urinary tract anomalies. Patient subsequently required gastrostomy tube for poor oro-motor function. She had serial casting of her feet for management of talipes equinovarus. Subsequently, she had two tenotomies. An MRI of the brain and spine done at 3 years of age showed 11 thoracic vertebrae with the conus medullaris being truncated and nodular at the level of intrapedicular T11. A rudimentary dysgenetic sacrum with a sagittal cleft in the median sacrum with rudimentary apophysis of both sides of the pelvis and a thickened cauda equine was detected. There was a sagittal cleft in the thecal sac extending from the level of the L4 pedicle. (Figure 5) On neurodevelopmental assessment at 18 and 24 months she had mild to moderate language, social, cognitive and fine motor deficits and moderate to severe motor deficit especially related to ambulation. Her last follow up was at the age of 3 years.

## 3. Discussion

CRS or CDS is also known as caudal dysplasia, sacral dysgenesis or regression, congenital sacral agenesis, sacral defect with anterior meningocele, sacro-coccygeal dysgenesis and caudal dysplasia sequence [1,2,3,4]. A related condition, sirenomelia sequence (mermaid syndrome) or symmelia, was thought to be a more severe form of CRS, but it is argued that it is a distinct entity [5,6]. Sirenomelia is characterized by fusion of the legs and a variable combination of the other abnormalities [5,6]. The prevalence of sirenomelia is about 1 in 100,000 live births [7]. The Currarino triad or sequence is a related, yet distinct condition and includes anorectal atresia or ectopia, coccygeal and partial sacral agenesis, and a pre-sacral mass lesion such as anterior meningocele, lipoma or dermoid cyst [8,9]. The prevalence of CRS in infants of diabetic mothers is documented at up to 1 in 350 live births and between 20% and 25% of mothers of infants with CDS have insulin-dependent diabetes mellitus [10,11].

Family history and maternal diabetes mellitus are two of the risk factors for this disorder. The abnormal embryologic development of the caudal mesoderm occurs within the first 4 weeks of embryonic development [12]. The exact pathogenesis of this syndrome is poorly understood, and several etiologic factors have been suggested. Maternal exposure to cocaine or alcohol consumption, is known to be associated in some cases. Other postulated mechanisms include vascular steal theory or hypo-perfusion, fetal hypoxemia and amino acid imbalances. A primary deficiency of the caudal mesoderm is implicated in CRS. In Sirenomelia vascular steal could play a more important role due to the presence of an aberrant umbilical artery (persistent vitelline artery) and the single midline lower limb. Genetic mutations in combination with environmental factors may lead to an increased risk of CRS. Multiple genetic factors such as mutations in the VANGL1 gene on chromosome 1p13, the CELSR1 and the HLXB9 gene on chromosome 7q36 in cases of the Currarino syndrome, have been implicated [13,14,15,16].

The clinical manifestations of this patient included bilateral clubfoot, anorectal malformation, and renal abnormalities including duplication of the collecting systems. CRS is a varied combination of abnormalities involving the musculoskeletal, gastrointestinal, genito-urinary and central nervous systems. The common factor to these abnormalities relates to defects in the development of the caudal mesoderm and the structures that it ultimately forms. Common findings include flexion contractures of the knees and hips, pelvic deformity, syn- or poly-dactyly, anorectal malformations, abdominal wall defects, gut malrotation, intestinal atresia, renal agenesis or dysplasia, absent bladder, transposition of external genitalia, hypospadias, myelomeningocele and hydrocephalus.

There are some differences in the classification of all CRS and sirenomelia. The Renshaw and Pang classification is used for the CRS, while the Pang, Kjaer and Stocker and Heifetz classification is used for sirenomelia [17,18,19,20]. Renshaw described CRS based on type of defect and articulation between bones. Type 1 has total or partial unilateral sacral agenesis; type II has variable lumbar and total sacral agenesis and the ilia articulates with the sides of the lowest vertebra; type III has variable lumbar and total sacral agenesis and the caudal end plate of the lowest vertebra rests above fused ilia or an iliac amphiarthrosis; type IV has fusion of soft tissues in both lower limbs; type V, also known as sirenomelia, has fused bones of lower limbs [20]. Stocker and Heifetz classified sirenomelia in 7 types: I would have all thigh and leg bones the type II has a single fibula, the type III has an absent fibulae, the type IV haspartially fused femurs and fused fibulae, the type V has partially fused femurs along with absent fibulae, the type VI has a single femur and a single tibia, and type VII has a single femur and absent tibiae [19]. After the second MRI, the patient described above has a CRS type II and this is based on her severe sacral dysgenesis with hypoplasia of the fifth lumbar vertebra.

Management of CRS is dependent on the specific anatomical abnormalities present in a given patient. This patient required surgical creation of a sigmoid colostomy, serial casting of her clubbed feet and bilateral tenotomy to release equinus contracture. Management of other CRS patients can include a wide variety of interventions to address the full spectrum of possible anatomical abnormalities. There are reports of the use of growth hormones with improved function by promoting distal innervation [21].

This spectrum of abnormalities across multiple organ systems although not an uncommon finding in cases of poorly controlled gestational diabetes, our patient presented with these malformations, consistent with CRS without a history of maternal diabetes. Understanding the varying complexity and multisystem involvement of this disease and rarity in nondiabetic mothers may allow for better multidisciplinary approach to management of such infants.
